# Two-step spatiotemporal anomaly detection corrected for lag reporting time with application to real-time dengue surveillance in Thailand

**DOI:** 10.1186/s12874-024-02141-5

**Published:** 2024-01-13

**Authors:** Chawarat Rotejanaprasert, Darin Areechokchai, Richard J. Maude

**Affiliations:** 1 Department of Tropical Hygiene, Faculty of Tropical Medicine, Mahidol University, Bangkok, Thailand; 2 Mahidol-Oxford Tropical Medicine Research Unit, Faculty of Tropical Medicine, Mahidol University, Bangkok, Thailand; 3 Division of Vector Borne Diseases, Department of Disease Control, Ministry of Public Health, Nonthaburi, Thailand; 4 Harvard T.H. Chan School of Public Health, Harvard University, Cambridge, MA USA; 5 Centre for Tropical Medicine and Global Health, Nuffield Department of Medicine, University of Oxford, Oxford, UK; 6 The Open University, Milton Keynes, UK

**Keywords:** Spatiotemporal, Dengue, Bayesian, Cluster detection, Delay

## Abstract

**Background:**

Dengue infection ranges from asymptomatic to severe and life-threatening, with no specific treatment available. Vector control is crucial for interrupting its transmission cycle. Accurate estimation of outbreak timing and location is essential for efficient resource allocation. Timely and reliable notification systems are necessary to monitor dengue incidence, including spatial and temporal distributions, to detect outbreaks promptly and implement effective control measures.

**Methods:**

We proposed an integrated two-step methodology for real-time spatiotemporal cluster detection, accounting for reporting delays. In the first step, we employed space-time nowcasting modeling to compensate for lags in the reporting system. Subsequently, anomaly detection methods were applied to assess adverse risks. To illustrate the effectiveness of these detection methods, we conducted a case study using weekly dengue surveillance data from Thailand.

**Results:**

The developed methodology demonstrated robust surveillance effectiveness. By combining space-time nowcasting modeling and anomaly detection, we achieved enhanced detection capabilities, accounting for reporting delays and identifying clusters of elevated risk in real-time. The case study in Thailand showcased the practical application of our methodology, enabling timely initiation of disease control activities.

**Conclusion:**

Our integrated two-step methodology provides a valuable approach for real-time spatiotemporal cluster detection in dengue surveillance. By addressing reporting delays and incorporating anomaly detection, it complements existing surveillance systems and forecasting efforts. Implementing this methodology can facilitate the timely initiation of disease control activities, contributing to more effective prevention and control strategies for dengue in Thailand and potentially other regions facing similar challenges.

**Supplementary Information:**

The online version contains supplementary material available at 10.1186/s12874-024-02141-5.

## Background

One of the most prevalent vector-borne infections is dengue fever with an estimated annual global burden of 390 million infections, of which 96 million present clinically [1]. The disease is caused by dengue virus principally transmitted by *Aedes* mosquitoes which are commonly found in tropical and sub-tropical regions. In addition, dengue has surpassed other infectious diseases such as malaria to be the most prominent vector-borne disease globally in terms of morbidity and cost of treatment [2]. The impact of dengue is a great burden on public health costs in South-East Asia and the burden of this infection in Thailand is among the highest in the world [3].

Dengue infection is commonly asymptomatic but when clinical manifestations occur, they can vary from mild to severe and life-threatening. Severe dengue, in particular dengue hemorrhagic fever (DHF) and dengue shock syndrome (DSS), is an important cause of hospitalization and death in Thailand [4]. The mild form of infection may be infectious and spread the virus in the community. The only available vaccine for dengue has limited efficacy and can only be administered to people who have previously been infected with challenges of pre-vaccination screening and suboptimal test performance [5]. Due to these limitations and the absence of any specific treatment, vector control has remained a focus of public health interventions to interrupt the infection cycle. Estimating when and where an outbreak will occur is an important goal to effectively allocate prevention and control resources. Therefore, efficient and reliable notification systems are vital to monitor dengue incidence including spatial and temporal distributions to detect outbreaks in order to initiate timely and effective control measures.

**Fig. 1:**
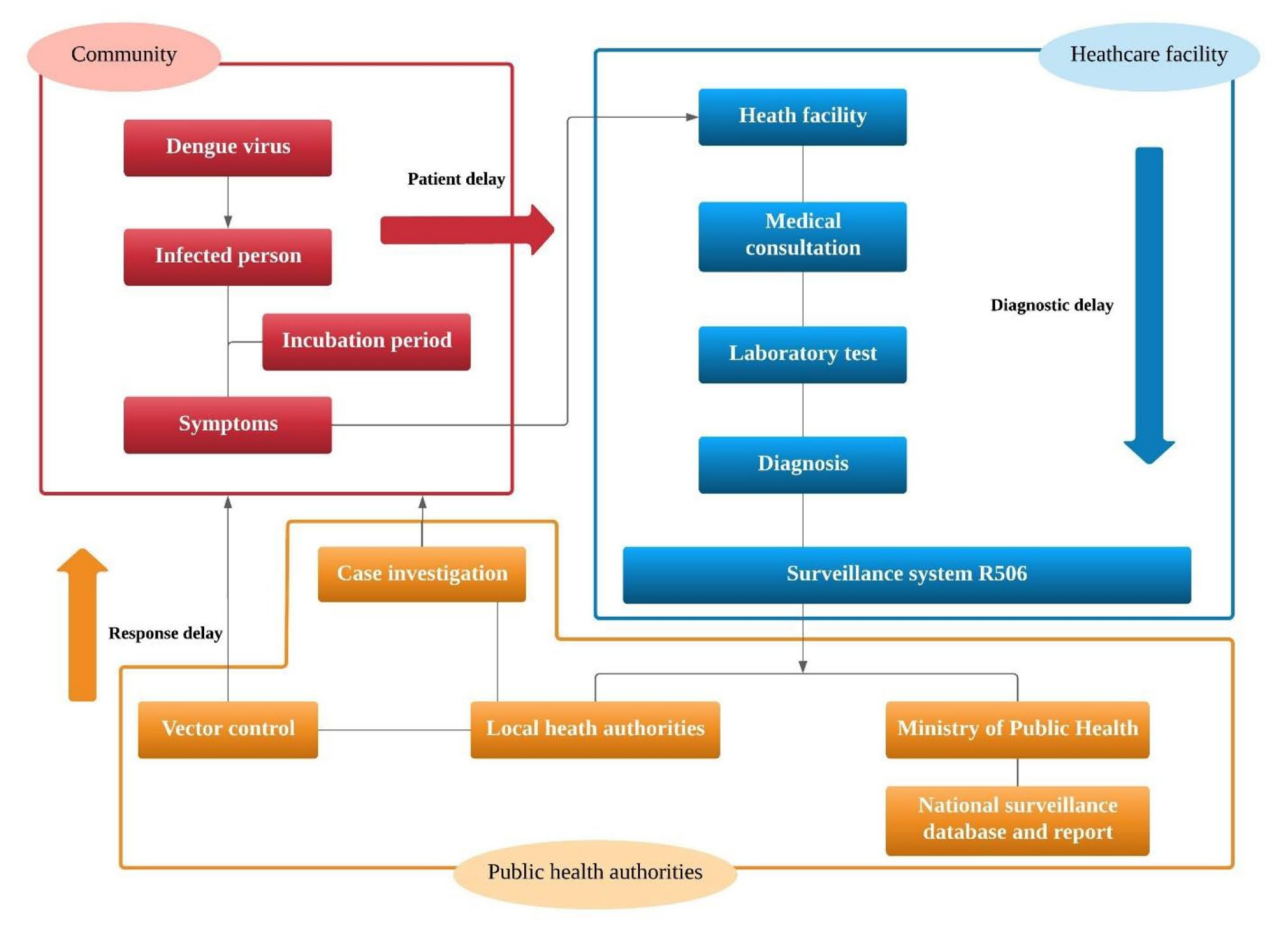
Flow chart of disease surveillance system with possible reporting delays in different parts of the system

Effective communicable disease surveillance systems are a prerequisite to ensure early detection of health threats and their timely control. Delay in infectious disease reporting might hamper timely outbreak interventions. In general, public health surveillance of diseases relies on the notification system which is a result of a chain of events from infection through reporting to public health services, be they local, regional or national. The general flow of surveillance information in Thailand is depicted in Fig. 1. Delays in the system arise at different stages: different health-seeking behaviors (community), laboratory and follow-up tests (health care facility), the reporting system, and communications between different health providers (surveillance response), including hospitals, the district officer and the insecticide sprayer operatives, as well as people in targeted areas. Dengue surveillance in many countries including Thailand relies on passive reporting which is susceptible to delays. The lag in the surveillance system is therefore a vital issue for disease control planning as incomplete and delayed information can undermine any efforts to deliver early warning and real-time outbreak detection required to trigger an effective response to public health threats.

Influenced by healthcare provider adherence and patient access, lagged reports exhibit variations across locations. Recent methodologies (examples [6–10]) aim to estimate current disease incidence by addressing notification lags, primarily focusing on systematic delays. However, these approaches overlook cluster detection, a crucial aspect in the decision-making process for disease outbreak control. While a prior effort offered a valuable framework for reporting delay correction in dengue control in Thailand [11], the correction alone falls short of the ultimate surveillance goal: informing public health actions to reduce morbidity and mortality [12]. Consequently, in this study, we went beyond delay correction, also implementing and comparing the performance of cluster detection methods with case nowcasting.

>

Reporting system time lags hinder timely cluster identification, impeding the initiation of effective disease control interventions. Therefore, we introduced an integrated two-step methodology for spatiotemporal real-time cluster detection, specifically tailored to correct reporting delays. The first step involved adopting space-time nowcasting modeling to account for reporting system lags. Subsequently, anomaly detection methods assessed adverse risks, demonstrated using weekly dengue surveillance data in Thailand. We also further evaluated effectiveness with various metrics compared different methods, revealing similarities and differences among detection techniques with optimal thresholds. This advancement offers valuable insights for informing additional public health actions to reduce dengue morbidity and mortality in Thailand.

## Methods

### Dengue surveillance data

In this study, we analyzed dengue case data obtained from the routine surveillance system of the Bureau of Epidemiology, Thai Ministry of Public Health. The dataset consisted of reported cases from various healthcare facilities, including governmental hospitals, clinics under the universal health coverage scheme, and private hospitals, all of which reported cases to district health surveillance data centers. To examine the influence of reporting delays and outbreaks, our study focused specifically on the data collected from the 50 districts of the Bangkok metropolitan area. The years 2010–2011 were chosen as they presented a significant and illustrative case study for our research objectives. During this period, widespread dengue outbreaks were observed across the country, with particular intensity in Bangkok. Notably, the response to these outbreaks exhibited notable delays. Therefore, this timeframe serves as a relevant case study to investigate the impact of reporting delays and outbreak occurrences.

**Fig. 2:**
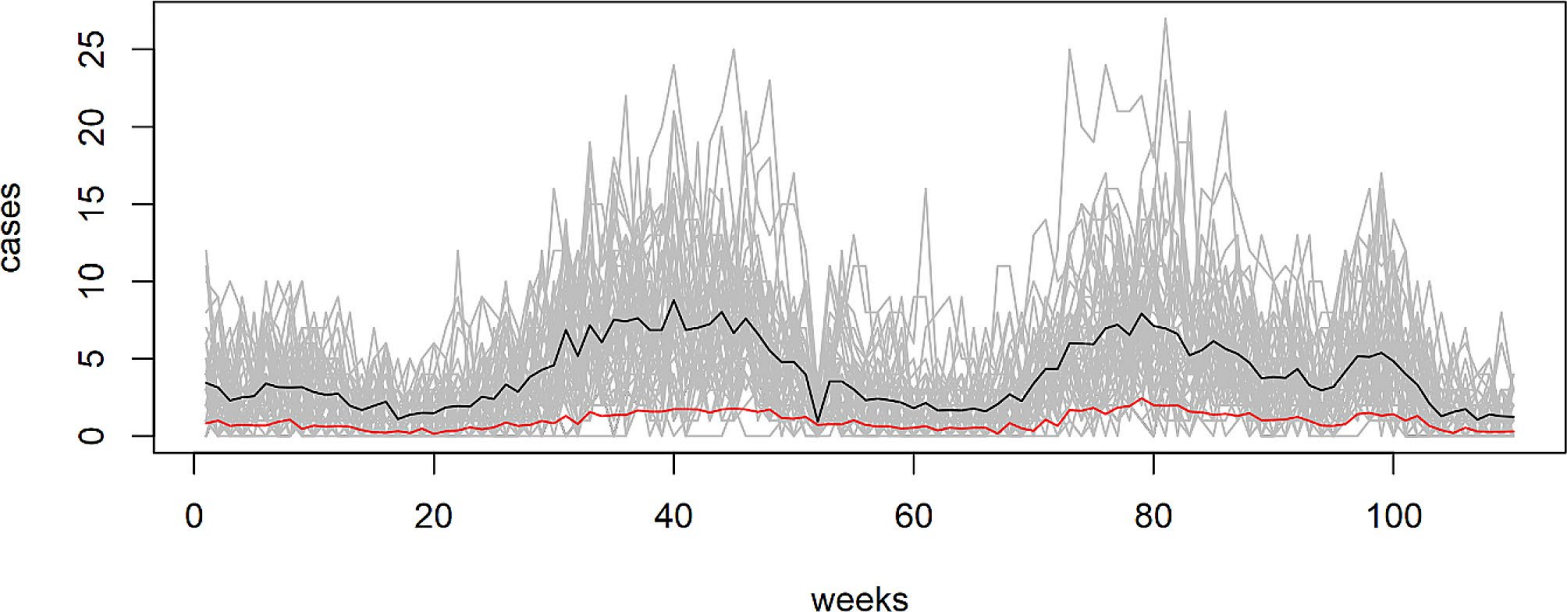
Plot of weekly dengue incidence in Bangkok, Thailand, 2010–2011. Grey lines represent reported dengue incidence for each district, while black and red lines depict true (no delays, black) and reported (with delays, red) dengue cases averaged over all districts for specific weeks

The dengue case types considered in our analysis encompassed dengue fever, dengue hemorrhagic fever, and dengue shock syndrome. Our primary goal was to achieve real-time detection, enabling prompt identification of dengue infection clusters and facilitating timely intervention to prevent further disease transmission. Consequently, we combined the number of cases across all dengue types in our analysis. Figure 2 illustrates the temporal trend of dengue incidence in Bangkok during the years 2010–2011. Notably, reporting delays tended to increase during the high season, which corresponds to the rainy period, potentially leading to substantial delays in the availability of data. Such delays can hinder the early detection of possible outbreaks, underscoring the significance of improving the timeliness of surveillance systems to enhance outbreak response capabilities.

### Ethics declarations

Ethics Committee of the Faculty of Tropical Medicine, Mahidol University waived for informed consent of participants. This study was approved by the Ethics Committee of the Faculty of Tropical Medicine, Mahidol University. The submission number was TMEC 22–054 and the number of ethical approval certificate was MUTM 2022-057-01. All methods were carried out in accordance with relevant guidelines and regulations.

### Nowcasting for lagged reporting

**Fig. 3:**
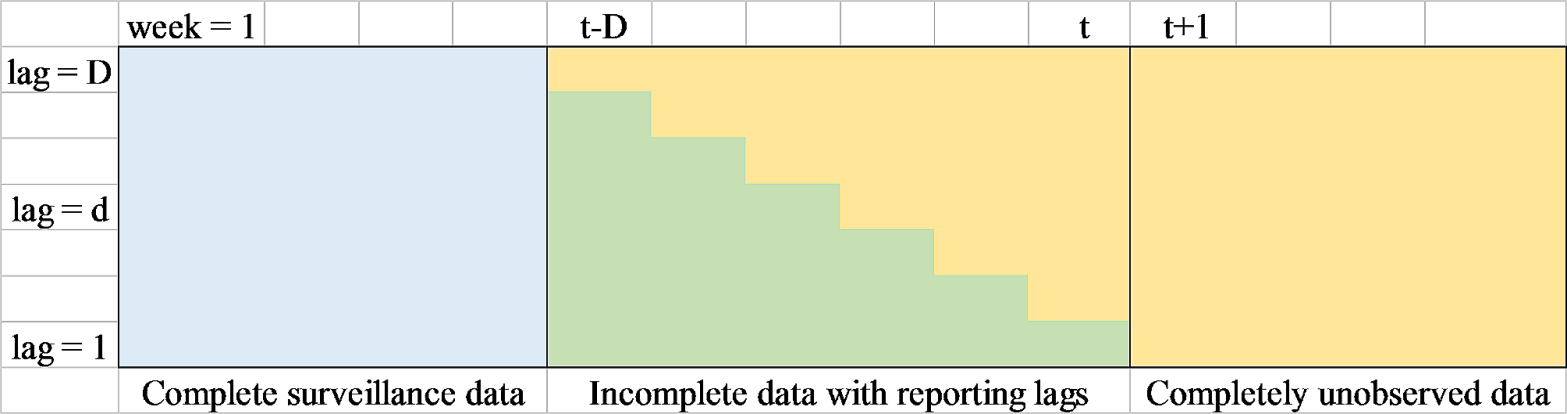
Surveillance reporting lag format. The blue cells represent completely observed data in the system for each district at week *t* and partially observed cases are in green cells. The yellow cells represent the unobserved data. *d* is the lag index with *D* maximum delays, i.e. delays beyond *D* were not considered

A key challenge for infectious disease surveillance in countries with developing infrastructure including Thailand is the time lag before reports are delivered at different levels in the notification system. The report structure of surveillance data with reporting lags can be seen as the lag triangle presented in Fig. 3. As described in [11], let ${y_{itd}}$ be the number of disease incidence which occurred during calendar week *t* in district *i* (*i* = 1,…, *I* = 50) but arrived in the surveillance database in week *d* (*d* = 1,…, *D*) weeks after the onset date. This signifies the problem that cases have been recorded but have not yet been entered into the database. Note that the event that the cases were in the surveillance system in the same week as the date of diagnosis was denoted as *d* = 1. The current time point of interest is indexed as *t* = T and the maximum possible delay that can happen in the surveillance system is labelled as *D*, i.e., full data were delivered into the system from *T* + *D* weeks onwards. Then$y_{{it}}^{*}=\sum\nolimits_{{d=1}}^{D} {{y_{itd}}}$can be defined as the estimated number of dengue cases that truly occurred by summing predicted reporting lag fractions happening at week *t*,${y_{itd}}$, over the possible lag range. The goal here was to correct the reported cases by nowcasting the actual weekly fractions of dengue cases for each district, $y_{{it}}^{*}$, in a real-time manner.

To address spatiotemporal reporting lags, a frequently adopted approach in small area health studies is to model case counts as conditionally independent Poisson variates. The likelihood function for this is defined as

1 $$f(y|\mu )=\frac{{{\mu ^y}}}{{y!}}\exp \left( { - \mu } \right)$$

where the mean and variance are both equal to$\mu$. That is, for our modeling, we assumed i.e.${y_{itd}}\sim Poisson({e_{it}}{\theta _{itd}})$where${\theta _{itd}}$was the relative dengue case risk adjusted for the offset,${e_{it}}$, as the baseline level at risk. There are a number of ways to adjust for the baseline (see examples [13–15]), however a common practice for disease mapping [16] is to calculate the expected rate as ${e_{it}}=\frac{{\sum\nolimits_{i} {\sum\nolimits_{t} {{n_{it}}} } }}{{\sum\nolimits_{i} {\sum\nolimits_{t} {po{p_{it}}} } }}po{p_{it}}$, where${n_{it}}$and$po{p_{it}}$are the true number of disease incidence and population at risk for each location and time. Since we performed the analysis at a weekly scale, the number of populations was assumed to be constant over the study period. Then the expected rate used in the analysis was computed as ${e_{it}}={e_i}=\frac{{\sum\nolimits_{i} {\sum\nolimits_{t} {{n_{it}}} } }}{{\sum\nolimits_{i} {\sum\nolimits_{t} {po{p_i}} } }}po{p_i}\,,\forall t$. Another main parameter of interest is ${\theta _{itd}}$and the most common approach to model this is to assume a logarithmic link to a linear combination of space-time random effects. First, we structured the model-based lag reporting correction by using information across neighboring districts and time periods to incorporate spatiotemporal smoothing. The convolution model (see examples [15–18]) was employed to capture spatially correlated and unstructured extra variation in the model. Both structured and unstructured random effects were included to capture various forms of unobserved confounding. The uncorrelated random effect is described by a zero mean Gaussian prior distribution. The spatially correlated effect is assumed to have the intrinsic conditional autoregressive model [19]. To capture the time series trend, the first-order random walk model was applied. All random interaction terms among space, time and delay dimension were specified by a Gaussian distribution with zero mean. All precision (reciprocal of variance) parameters were assumed as a Log-Gamma distribution with hyperparameters as 1 and 0.0005, and 1 and 0.00005 for the conditional autoregressive model, and for uncorrelated and random walk random effects.

To address the variability in dengue incidence, the Negative Binomial distribution, which incorporates an overdispersion parameter, can be considered as an alternative to the Poisson likelihood. Typically, issues of dispersion can be tackled through models like Negative Binomial and Quasi-Poisson, both having an equal number of parameters and suitability for overdispersed count data [20]. In our exploration of modeling choices for reporting lags in this study, we also considered the Generalized Poisson model as an alternative base count distribution. This model not only accommodates dispersion but also possesses a heavier tail with the same first two moments, offering increased flexibility for a broader range of data compared to the Negative Binomial [21].

The Generalized Poisson model can be seen as a characterization, operating as an alternative Poisson mixture model to the Negative Binomial distribution for overdispersed count data, as emphasized in a study cited in our original submission [21]. Moreover, another study suggests that generalized Poisson regression models can serve as viable alternatives to negative binomial regression [22]. Despite the typical preference for the Negative Binomial distribution when evidence of dispersion is present relative to the Poisson, a Negative Binomial model had previously shown similar performance to the Poisson in a scenario involving delay correction with mild overdispersion [11]. Additionally, during our extended study period, we noted similarities in temporal patterns and magnitudes compared to the previous study period. Consequently, we chose to compare only Poisson and Generalized Poisson models in this study.

The generalized Poisson distribution used in this study follows the form introduced in previous works [23, 24], represented as

2 $$f(y|\mu ,\varphi ,\delta )=\frac{{\mu {{(\mu +\varphi {\mu ^{\delta - 1}}y)}^{y - 1}}}}{{{{(1+\varphi {\mu ^{\delta - 1}})}^y}y!}}\exp \left( { - \frac{{\mu +\varphi {\mu ^{\delta - 1}}y}}{{1+\varphi {\mu ^{\delta - 1}}}}} \right)$$.

Given$\delta =1$, we have the mean and variance equal to$\mu$and$\mu {(1+\varphi )^2},\varphi >0$. When$\varphi \to 0$, the generalized Poisson approaches the Poisson distribution with mean and variance equal to$\mu$. The mean is also linked to the linear predictor with the logarithm function as in the Poisson.

### Space-time cluster diagnostics

Space-time cluster diagnostics in epidemiology often employ scan statistics and various refinements of scan statistics have been proposed (for example [25–27]), including the version implemented in SatScan software [28]. However, a fundamental challenge lies in interpreting p-values and establishing a threshold for defining ‘significance’ [29]. Therefore, we alternatively based our approaches in this study to cluster detection within the model-based framework.

In the context of this framework, it becomes crucial to define what constitutes a cluster. In infectious disease surveillance, it is important to effectively identify localized case anomaly that deviate from expected baseline patterns in both space and time, prompting further investigation. This concept is akin to anomaly detection, where we employ the goodness of fit of a model to quantify unusual events within a set of space-time observations. Measures of goodness of fit help summarize the differences between observed local case counts and the values expected under the model or baseline for each location and time. In our study, we thus explored and compared various model-based measures for anomaly detection, including exceedance probability, information criteria, and leave-one-out cross-validation.

#### Exceedance probability

A number of diagnostic tools are available to evaluate the local anomalies. However, it is a natural idea to consider a cluster as any isolated locations or geographically-bounded regions that display an excess of disease risk or incidence in a particular time. The excess of disease risk can be examined by comparison with the expected rate previously described. So, an approach for space-time anomaly detection is to calculate $P({\theta _{it}}>a)$, exceedance probability (EXC), from the number of estimates in the posterior sample which exceed a threshold [30, 31]. Usually the limit is assumed to be *a*= 1 which means we apply the level of the expected rate as the baseline.

#### Information criteria

An aim of diagnostic checking is to compare observed data with the fitted model in such a way that it is possible to detect any discrepancies. Forms of model assessment involve measuring the goodness-of-fit (GOF) to evaluate whether the particular data in space and time provide an adequate fit to the model. A set of common GOF measures is the information criteria. The deviance information criterion (DIC) [32] has been widely used for overall model fit in the Bayesian setting generalized from the Akaike information criterion (AIC) in the Frequentist framework. Another is the widely applicable or Watanabe-Akaike information criterion (WAIC) [33] which can be viewed as an improvement on DIC. WAIC is fully Bayesian in which this measure applies the entire use of posterior distribution. Unlike DIC, WAIC is robust to different parametrizations and is also valid for singular models [34].

While the global information criteria have been primarily used as an overall measure of model fit, they can be partitioned into contributions from individual observations in space and time to provide finer details of model discrepancies [35, 36]. The partitioning of the DIC for the observed data, local DIC, can be written as $DI{C_{it}}=\bar {D}({\varvec{\theta}_{it}})+p{D_{it}}$ [36] where$\bar {D}({\varvec{\theta}_{it}})$is the mean deviance for nowcasted cases at district *i* and week *t* and $p{D_{it}}$is the effective number of parameters, amount of information used for the particular observation for each location and time. Likewise, local WAIC, which is a direct result of pointwise predictive density, can be defined as $WAI{C_{it}}=lpp{d_{it}}+pWAI{C_{it}}$ [34] where $lpp{d_{it}}$(log pointwise predictive density) = $- 2\log \left( {\bar {f}(y{*_{it}}|{\varvec{\theta}_{it}})} \right)$and$pWAI{C_{it}}=2\operatorname{var} \left( {\log \left( {f(y{*_{it}}|{\varvec{\theta}_{it}})} \right)} \right)$calculated over the posterior sample. Since the range of information criteria is on the positive real line, we adopted the transformed values on a unit interval as 1-${e^{ - DIC}}$and 1- ${e^{ - WAIC}}$. This similar transformation was also utilized as model probability in model selection and averaging [36, 37].

#### Leave-one-out cross-validation

Another set of metrics widely used to estimate the model fit error is cross validation. In a general setting of cross-validation, the data are repeatedly divided into a training set and a test set. Then the model is fitted using the training set and the cross-validation error is calculated from the test set. However, we restricted our attention here to leave-one-out cross-validation (LOO-CV), the special case with all partitions in which each test set represents a single data point. Among LOO-CV methods, the conditional predictive ordinate (CPO) [38] and probability integral transform (PIT) [39] are commonly used to detect extreme observations in statistical modeling. The CPO detection in our case for the delay-corrected dengue incidence at district *i* during week *t* can be computed as $CP{O_{it}}=\int {f(y{*_{it}}|} {\varvec{y}}{*_{ - it}},{\varvec{\theta}_{it}})\pi ({\varvec{\theta}_{it}}|{\varvec{y}}{*_{ - it}})d{\varvec{\theta}_{it}}$. For each observed case, its CPO is the posterior probability of observing that dengue case when the model is fit using all data except $y{*_{it}}$. Large CPO values imply a good fit of the model to observed data, while small values suggest a worse fitting of the model to that observed data point and, perhaps, that it should be further explored.

On the other hand, PIT measures the probability of a new value to be less than the actual observed value:$PIT{}_{{it}}=\pi (y_{{it}}^{{new}} \leqslant {y_{it}}|{{\varvec{y}}_{ - it}})$ where ${{\varvec{y}}_{ - it}}$is the observation vector with the *it-*th component omitted. This procedure is performed in cross-validation mode meaning that in each step of the validation process the ensuing leave-one-out posterior predictive distribution is calculated. However, in our data which are discrete (disease count) data, the estimate was adjusted as$PIT_{{it}}^{{adjust}}=PI{T_{it}} - 0.5 \times CP{O_{it}}$, and unusually large or small values of PIT indicate possible outliers or surprising observations not supported by the model under consideration [40].

#### Evaluation and computation of anomaly diagnostic methods

Surveillance systems for infectious diseases must strike a balance between outbreak detection accuracy and the efficient allocation of disease control resources. The concepts of optimal criteria, accuracy (Acc), sensitivity (Se), specificity (Sp), positive predictive value (PPV), and negative predictive value (NPV) serve as valuable metrics for comparing and assessing the validity of cluster detection methods. In this study, these five evaluation metrics were employed for method comparison and performance evaluation. An anomaly was considered alarmed when the anomaly diagnostic value from space-time cluster diagnostics, computed for each case count, exceeded a predefined cutoff. We then systematically evaluated the performance of the cluster diagnostics across different threshold values.

The key evaluation components are defined as follows. The true positive (TP) was calculated as instances where a method correctly indicates the presence of a disease anomaly. True negative (TN) was the count where a method correctly indicates the absence of a disease anomaly. False positive (FP) was the count of cases where a method incorrectly suggests the presence of an anomaly. False negative (FN) was the count of instances where a method incorrectly indicates the absence of an anomaly. Then sensitivity, specificity, and predictive values are expressed as follows: sensitivity = TP / (TP + FN); specificity = TN / (FP + TN); positive predictive value = TP / (TP + FP); negative predictive value = TN / (TN + FN); accuracy is defined as the proportion of correct detections among the total number of detections, i.e., Acc = (TP + TN) / (TP + TN + FP + FN).

In order to efficiently apply this methodology in real surveillance situations, one essential characteristic that should be considered in real-time surveillance systems is computational practicability. Using all the data history is perhaps unnecessary while the most recent information might be adequate to capture the disease pattern needed to detect an outbreak. To reduce computing resource, we partitioned the surveillance data into sliding windows to optimize computational competence of the system. Rather than the full likelihood, the working likelihood was partitioned as $\Pi _{t = T - w + 1}^T\Pi _{d = 1}^D\Pi _{i = 1}^If({y_{itd}}|{\theta _{itd}})$where *w* is the length of sliding window. The sliding window technique then investigates only the most recent *w* and hence the surveillance might be more efficient and practical for real-time applications. However, the partition can be a trade-off between computing efficiency and estimation of precision. We then also examined the effect of different window sizes in the case study.

Estimates derived from the models and diagnostic methods are typically computed from converged posterior samples using sampling-based algorithms like Markov Chain Monte Carlo (McMC). However, real-time estimation in infectious disease surveillance requires timeliness. With the setup of a multidimensional model and accumulating surveillance data over time, the parameter space can rapidly expand, demanding exponential computational resources. To address this, a more efficient approach for inferring parameters is the Integrated Nested Laplace Approximation (INLA) [41]. This method is particularly suitable for the rapid estimation of parameters in a real-time context. The proposed model was implemented using the numerical Laplace approximation within the R-INLA package, available at www.r-inla.org. All computations were conducted using RStudio version 2020.07.0. Computing information using INLA with R code was provided in supplementary document S1.

## Results

The data employed to demonstrate anomaly detection consisted of weekly dengue incidence in Bangkok, the location with the highest annual incidence in the country. Results, averaged across study areas and detection thresholds, are presented in Table 1, detailing estimates of sensitivity, specificity, accuracy, and their corresponding predictive values for anomaly detection. Without delay correction, the accuracy of detection methods under both likelihood assumptions ranged from 0.4791 using PIT to 0.6092 using WAIC. DIC and EXC performed best under a General Poisson model while WAIC and EXC had the best outcome with a Poisson model. The highest accuracy with reporting delay was the Poisson model with WAIC. With nowcasting correcting for reporting lags, EXC performed best across the evaluation metrics with accuracies of 0.7221 and 0.6916 under both Poisson and Generalized Poisson models. The accuracies with corrected delays using the proposed spatiotemporal nowcasting technique were improved about 22.7% and 17.52% under Poisson and Generalized Poisson assumptions respectively.

**Table 1:** Comparison of model-based cluster detection methods with and without nowcasting for reporting lags under evaluation metrics and likelihood assumptions. The bold numbers represent the highest value in each category

Likelihood	Delay	Cluster	Evaluation metric				
model	correction	detection	Se	Sp	NPV	PPV	Acc
		EXC	**0.8723**	**0.6123**	**0.8394**	**0.6531**	**0.7221**
		CPO	0.8241	0.2324	0.6895	0.4863	0.5237
	Yes	PIT	0.4025	0.5579	0.5291	0.4308	0.4874
		DIC	0.8611	0.2313	0.6671	0.4822	0.5172
		WAIC	0.8661	0.2326	0.6929	0.4869	0.5247
Poisson		EXC	0.1135	**0.9833**	0.5716	**0.8502**	0.5885
		CPO	0.7063	0.4141	**0.7201**	0.5336	0.5921
	No	PIT	0.4146	0.5804	0.5439	0.4511	0.5051
		DIC	0.7562	0.4672	0.6975	0.5413	0.5984
		WAIC	**0.7778**	0.4689	0.7175	0.5491	**0.6092**
		EXC	**0.8611**	**0.5021**	**0.8291**	**0.6296**	**0.6916**
		CPO	0.8477	0.1951	0.6467	0.5116	0.5325
	Yes	PIT	0.3862	0.4337	0.4779	0.4403	0.4618
		DIC	0.8589	0.1928	0.6464	0.5112	0.5319
Generalized		WAIC	0.8577	0.1951	0.6466	0.5116	0.5325
Poisson		EXC	0.0161	**0.9889**	0.5716	**0.8502**	0.5885
		CPO	0.7771	0.4281	0.6691	0.5634	0.5981
	No	PIT	0.3129	0.6369	0.4939	0.4501	0.4791
		DIC	0.7777	0.4286	**0.6699**	0.5638	**0.5987**
		WAIC	**0.7779**	0.4281	0.6691	0.5634	0.5981

**Fig. 4:**
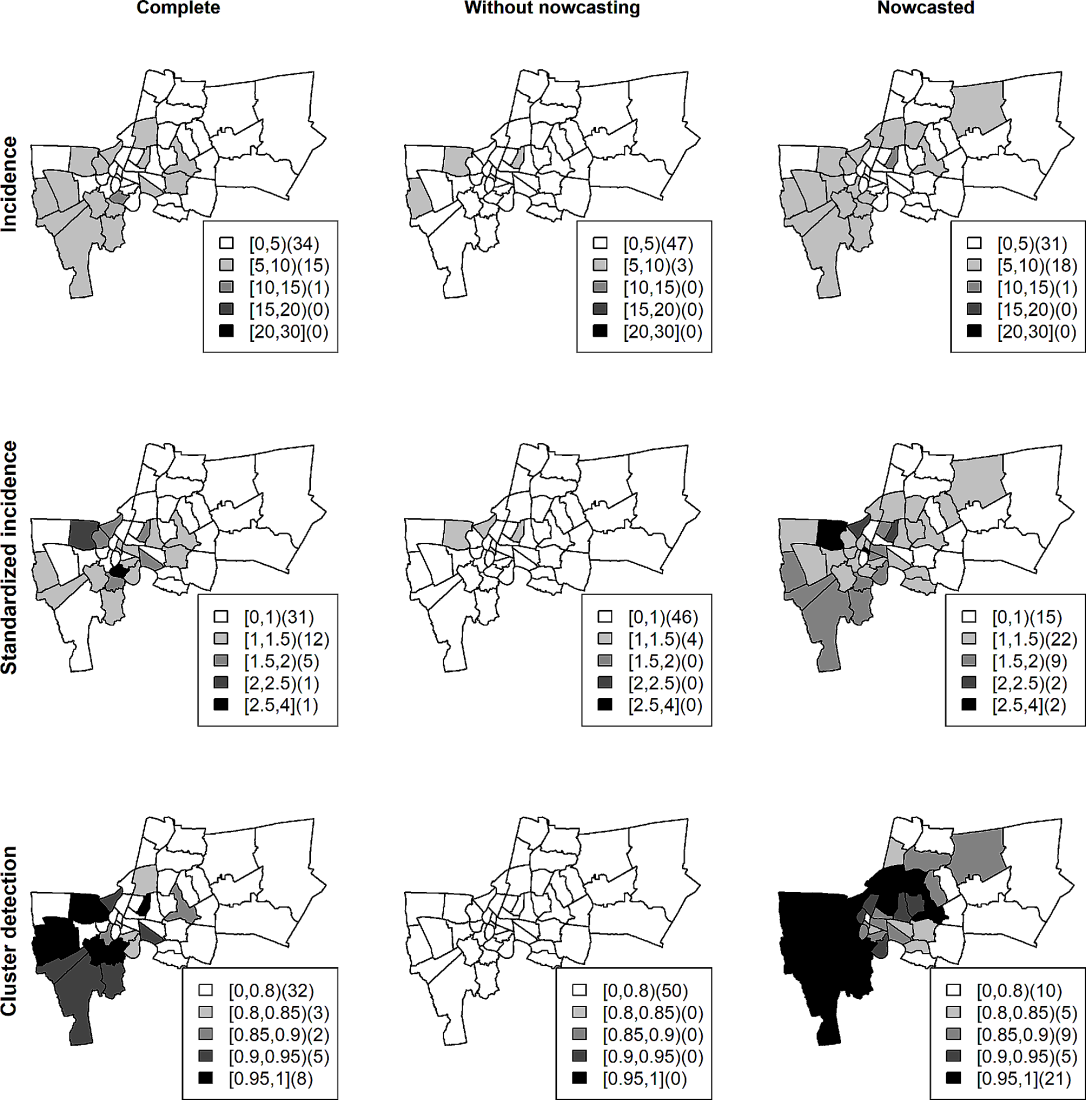
Maps of crude incidence (top row), standardized incidence (middle row), and cluster detection (bottom row) using exceedance probability of complete dengue reported cases in Bangkok. Left column: data with nowcasting. Middle column: data without nowcasting. Right column: data with nowcasting during week 102 of the study period

**Table 2:** Detection characteristics and parameters with different sliding window sizes and likelihood assumptions

Likelihood	Detection	Window size (weeks)						
model	characteristic	5	10	15	20	25	30	
	Max accuracy	0.7143	0.7214	0.7175	0.7234	0.7209	0.7202	
Poisson	Cut-off (percentile)	0.97	0.98	0.95	0.93	0.98	0.98	
	Time	0.5376	2.1435	6.5293	14.6618	28.5325	48.6852	
	Max accuracy	0.7081	0.7158	0.7145	0.7214	0.7227	0.7222	
	Cut-off (percentile)	0.93	0.95	0.94	0.95	0.98	0.98	
Generalized	Time (min)	0.5487	2.2986	6.7418	15.8412	30.8942	53.2669	
Poisson	Overdispersion delay	0.0862	0.0848	0.0937	0.0861	0.0923	0.0918	
	95% CrI	(0.041, 0.124)	(0.051, 0.135)	(0.055, 0.167)	(0.094, 0.191)	(0.092, 0.148)	(0.091, 0.129)	
	Overdispersion cluster	0.158	0.1636	0.1551	0.1534	0.1478	0.1466	
	95% CrI	(0.045, 0.374)	(0.046, 0.384)	(0.042, 0.371)	(0.042, 0.364)	(0.042, 0.353)	(0.041, 0.351)	

We further examined the optimal threshold and effect of different window sizes in order to apply the cluster detection in real situations. The focus was limited to the test characteristics of EXC since the detection had the best performance across the evaluation measures and likelihood assumptions. The best threshold was defined as the cut-off value with the maximum accuracy. Table 2 shows the cut-off points with the highest accuracy using different computing window lengths. These comparisons were computed on a Dell computer with 64-bit Windows system, 8GB RAM and Intel i5-3570 S CPU @ 3.10 GHz. The optimal threshold varied in a range of 0.95–0.99 for Poisson and 0.93–0.99 for Generalized Poisson models with the maximum accuracy of approximately 72%. The computing times ranged from 0.5376 min per calculation with 5-week window size to 48.6852 min per calculation with 30-week window size under Poisson model, however the accuracy increased less than 1%. On the other hand, the Generalized Poisson model required slightly more computing time of 0.5487 min for 5-week and 53.2669 min for 30-week window sizes. The improved accuracy was also similarly small at less than 1%. The posterior summary of overdispersion parameters with their corresponding credible intervals (CrI) for both delay correction and anomaly detection indicated a mild overdispersion in the observed data with posterior means of 0.0861–0.0937 (95% CrI: 0.041–0.167) and 0.1466–0.1636 (95% CrI: 0.041–0.384). These implied that the Poisson likelihood assumption with space-time random effects might be adequate to capture the case variability in our data set.

Figure 4 compares dengue incidence, standardized incidence and exceedance probability at week 102 during the high season in year 2011. Note that the result of other periods (weeks 96–104) was provided in supplementary document S2. The complete (true) incidence depicted in the left column showed a possible disease cluster in the southwest of Bangkok and hot spots in the center. Exceedance probabilities also revealed the same pattern of high-risk areas using complete and nowcasted data. In contrast, those clusters and hot spots did not appear in data with reporting delays. The reporting lags are crucial for infectious disease surveillance as the infection can actually spread during the lag period while anomaly detection with nowcasting could accurately recover and detect potential outbreaks in the case study. The developed methodology hence demonstrated an advantage in revealing the true disease pattern properly for real-time public health intervention planning.

## Discussion

Efficient surveillance is paramount for early infectious disease outbreak detection, particularly for diseases like dengue with no effective vaccines or specific treatments. As vector control remains the primary intervention, timely outbreak detection is crucial. In this study, we devised an integrated approach to assess risks while addressing reporting lags, comparing anomaly detection measures in a dengue surveillance case study in Thailand. Unlike prior efforts that often focus solely on delay correction, we extended our investigation to include and compare cluster detection methods, augmenting the decision-making process for disease outbreak control.

Spatiotemporal cluster detection typically necessitates complex models, especially when modeling specific localized space-time behaviors. Real-time infectious disease surveillance requires effective clustering methods capable of promptly detecting deviations from normal background variation. To accommodate space-time reporting variations, we modeled dengue case counts using a count likelihood with a spatiotemporal latent random-effect structure. While a Poisson distribution is a common choice, our investigation also included a Generalized Poisson assumption, offering flexibility for a wider range of data compared to the negative binomial [21].

The dispersion parameter, indicative of data variability, demonstrated mild dispersion across scenarios and window sizes. The use of a Generalized Poisson model, known for its flexibility in handling dispersion, proved effective in capturing complex multidimensional correlations, though at the expense of increased computing time. Considering the real-time surveillance context, the feasibility of model computation should be a key consideration. Experiments with different moving window lengths revealed marginal improvements in accuracy, suggesting that small sliding windows can yield reasonably good performance, capturing data variation adequately within the model specification.

A number of measures of adverse risks were compared and investigated. The exceedance probability outperformed followed by information criteria and leave-one-out cross validation. PIT had the lowest overall performance but higher specificity than information criteria. Information criteria and CPO appeared to have high sensitivity but low PPV. This may imply that PIT yielded conservative detection while CPO and information criteria may produce more false positives. EXC appeared to have highest specificity and PPV without lag nowcasting and had the best values across evaluation metrics with correction for delays. Although WAIC has been suggested lately as an alternative to DIC, which has a long historical development in Bayesian statistics, in our case study both WAIC and DIC had very similar results and performance in various assessment measures. The choice of the most appropriate measure should consider the specific requirements and objectives of the surveillance system.

Timeliness is a critical aspect of real-time surveillance. One of the key advantages of our proposed framework is its minimal data requirement, as it solely relies on past surveillance data on incidence reporting using a sliding window partition. This flexibility allows the system to be readily adaptable to various disease systems, particularly in cases where other variables such as climatic or clinical confounders are not available in real-time for inclusion in the model. Nevertheless, our unified approach has been designed to accommodate the inclusion of such covariates through the link function, providing a comprehensive framework for capturing additional factors.

Despite its advancements, it is important to acknowledge several limitations in this study. Firstly, the developed methodology does not explicitly include prediction, which is a significant aspect of disease surveillance and planning. However, to support real-time disease control activities, our development effectively complements existing disease prediction efforts. The incorporation of lag-corrected nowcasting into forecasting can enhance the effectiveness of surveillance in disease control activities.

Another limitation is the exclusive testing of the developed platform using dengue data from Thailand. Generalizing its applicability to other diseases and settings may require further validation. Nevertheless, the developed platform demonstrates potential for a broad spectrum of applications, extending beyond dengue clustering scenarios to address challenges in infectious or emerging disease surveillance. The versatility and robustness of our approach render it applicable to various disease surveillance problems, providing public health practitioners with an effective tool for enhancing real-time monitoring, control, and prediction of infectious diseases.

## Conclusions

Effective disease surveillance systems are crucial for timely detection and control of health threats. However, reporting lags in infectious disease surveillance systems can hinder the prompt implementation of outbreak control measures. Existing methods for estimating disease incidence often overlook anomaly detection in the presence of reporting delays. In this study, we introduced an integrated approach that addresses this challenge by enabling accurate real-time cluster detection, even in the presence of reporting delays. While further research and collaboration are necessary to enhance the methodology and its development, our approach offers flexibility by relaxing disease-specific assumptions, making it adaptable to various disease settings. By incorporating anomaly detection, our method can effectively identify disease clusters in real-time, contributing to timely initiation of disease control activities. Furthermore, the efforts made in this study can complement existing surveillance systems and forecasting methods. By integrating our approach into the existing infrastructure, we can enhance the overall surveillance effectiveness and facilitate the timely implementation of disease control measures.

### Electronic supplementary material

Below is the link to the electronic supplementary material.

Supplementary Material 1

## Data Availability

The data that support the findings of this study were obtained from the Thai Bureau of Epidemiology, Ministry of Public Health, but restrictions apply to the availability of these data, which were used with permission for the current study, and are therefore not publicly available. For data requests related to this study, please contact the corresponding author, Dr. Chawarat Rotejanaprasert, at chawarat.rot@mahidol.ac.th. Data may be available from the authors upon a reasonable request and with permission of the Thai Bureau of Epidemiology.

## Abbreviations

DIC: Deviance Information Criterion
EXC: Exceedance probability
CPO: Conditional predictive ordinate
PIT: Probability integral transform
WAIC: Watanabe-Akaike information criterion
CrI: Credible interval
TP: True positive
TN: True negative
FP: False positives
FN: False negatives
Se: Sensitivity
Sp: Specificity
PPV: Positive predictive value
NPV: Negative predictive value
Acc: Accuracy
