# Supplementary material for: Two-step spatiotemporal anomaly detection corrected for lag reporting time with application to real-time dengue surveillance in Thailand
Source: BMC Med Res Methodol. 2024 Jan 13;24:10. doi: 10.1186/s12874-024-02141-5 (PMC10787994; doi:10.1186/s12874-024-02141-5)
Supplement: Supplementary file 1 — Supplementary Material 1 [file 12874_2024_2141_MOESM1_ESM.docx]

**Supplementary document: Two-step spatiotemporal anomaly detection corrected for lag reporting time with application to real-time dengue surveillance in Thailand**

**Supplementary document S1:** **Model computing information with R code**

In the context described in the main text, dengue cases occur weekly but may undergo reporting delays, as demonstrated earlier. Estimating the true number of cases involves approximating the weekly underreported cases for each district due to these delays. From a Bayesian standpoint, these delays can be treated as missing data. One approach to impute these missing values is by leveraging the posterior predictive distribution, which enables the computation of both point estimates and associated uncertainty. The posterior predictive distribution of delays is given by equation (S1):

$p(y_{itd}|y_{w})=\int_{\Theta} p(y_{itd}|\Theta_{w},y_{w})p(\Theta_{w}|y_{w})d\Theta_{w}$ (S1)

where$y_{w}$ and $\Theta_{w}$ refer to the data and parameters in the sliding window with a length *w* used to fit the model.

Estimates from the models and diagnostic methods are typically computed from converged posterior samples using sampling-based algorithms like Markov Chain Monte Carlo (MCMC). However, real-time estimation in infectious disease surveillance necessitates timeliness. With the establishment of a multidimensional model and the accumulation of surveillance data over time, the parameter space can rapidly expand, requiring exponential computational resources. To address this challenge, a more efficient approach for inferring parameters is the Integrated Nested Laplace Approximation (INLA) [1]. This method is particularly suitable for the swift estimation of parameters in a real-time context. The proposed model was implemented using the numerical Laplace approximation within the R-INLA package, accessible at www.r-inla.org.

As prediction involves fitting a model with missing data, we need to set the response variables as "NA" for those observations from which we intend to obtain estimates from the predictive distribution [2, 3]. Assuming we have the real-time surveillance reporting delay structure, as illustrated in figure 3 in the main text, the implementation and computation of estimated cases and anomaly detection corrected for reporting delays in our application can be executed using the following R-INLA code:

#Set up the model

t0 <- Sys.time()

for(mw in 1:wl){

w <- ws+ws*(mw-1)

t1 <- wl*ws

for(t in t1:t2){

n = array(rep(0,D*t*I),dim = c(D,I,t))

n <- dis_delay_true[,,(t-(w-1)):t]

for(i in 1:I){

x<-n[,i,]

x[t(apply(lower.tri(n[,i,]),1,rev))]<-NA

n[,i,]<-x

}

ID.DIT <- seq(1,D*I*w)

ID.D <- rep(seq(1,D),w*I)

ID.DI <- rep(seq(1,D*I),w)

ID.T <- numeric(0)

ID.DT <- numeric(0)

ID.I <- numeric(0)

ID.IT <- numeric(0)

Ey <- numeric(0)

j <- 1

for(ww in 1:w){

ID.T <- append(ID.T,rep(ww,D*I))

ID.DT <- append(ID.DT,rep(seq((ww-1)*D+1,ww*D),I))

for(i in 1:I){

ID.I <- append(ID.I,rep(i,D))

ID.IT <- append(ID.IT,rep(j,D))

j <- j+1

Ey <- append(Ey,rep(E[i,1],D))

}

}

data <- data.frame(y = as.vector(n), E=Ey, ID.I = ID.I, ID.T = ID.T, ID.D = ID.D, ID.DI = ID.DI, ID.DT = ID.DT, ID.IT = ID.IT, ID.DIT = ID.DIT)

f <- y ~ 1+f(ID.I,model="bym",graph=dis.bkk.adj)+f(ID.T,model="rw1")+f(ID.D,model="rw1")+f(ID.IT,model="iid")+f(ID.DT,model="iid")+f(ID.DI,model="iid")+f(ID.DIT,model="iid")

#This is for the Generalized Poisson model. However, it can be changed to other likelihood #functions

mod.delay <- inla(f,family="gpoisson",data=data,control.compute=list(waic=TRUE,dic=TRUE,cpo=TRUE))

a.U = array(mod.delay$summary.linear.predictor[,5],dim=c(D,I,T))

a.L = array(mod.delay$summary.linear.predictor[,3],dim=c(D,I,T))

a = array(mod.delay$summary.linear.predictor[,1],dim=c(D,I,T))

print(t)

print(w)

}

t00 <- Sys.time()

cpu.time[mw] <- t00-t0

print(mw)

}

**Supplementary document S2: Maps of dengue incidence, standardized incidence and cluster detection using exceedance probability (EXC) and true cases, with and without nowcasting (delay correction) during weeks 96-104.**

**
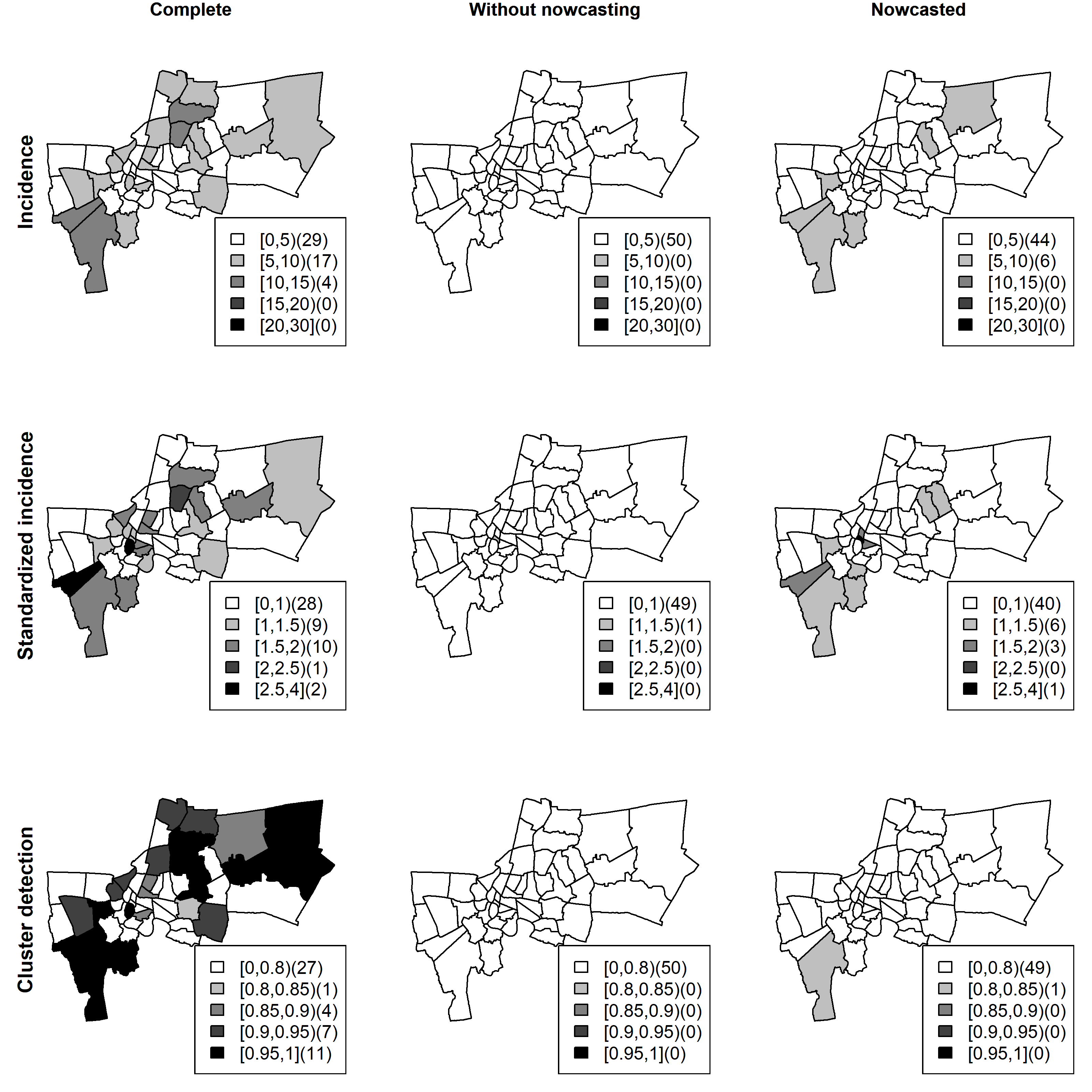
**

**Fig S1** Maps of dengue incidence, standardized incidence and cluster detection using EXC and true cases, with and without nowcasting during week 96.

**
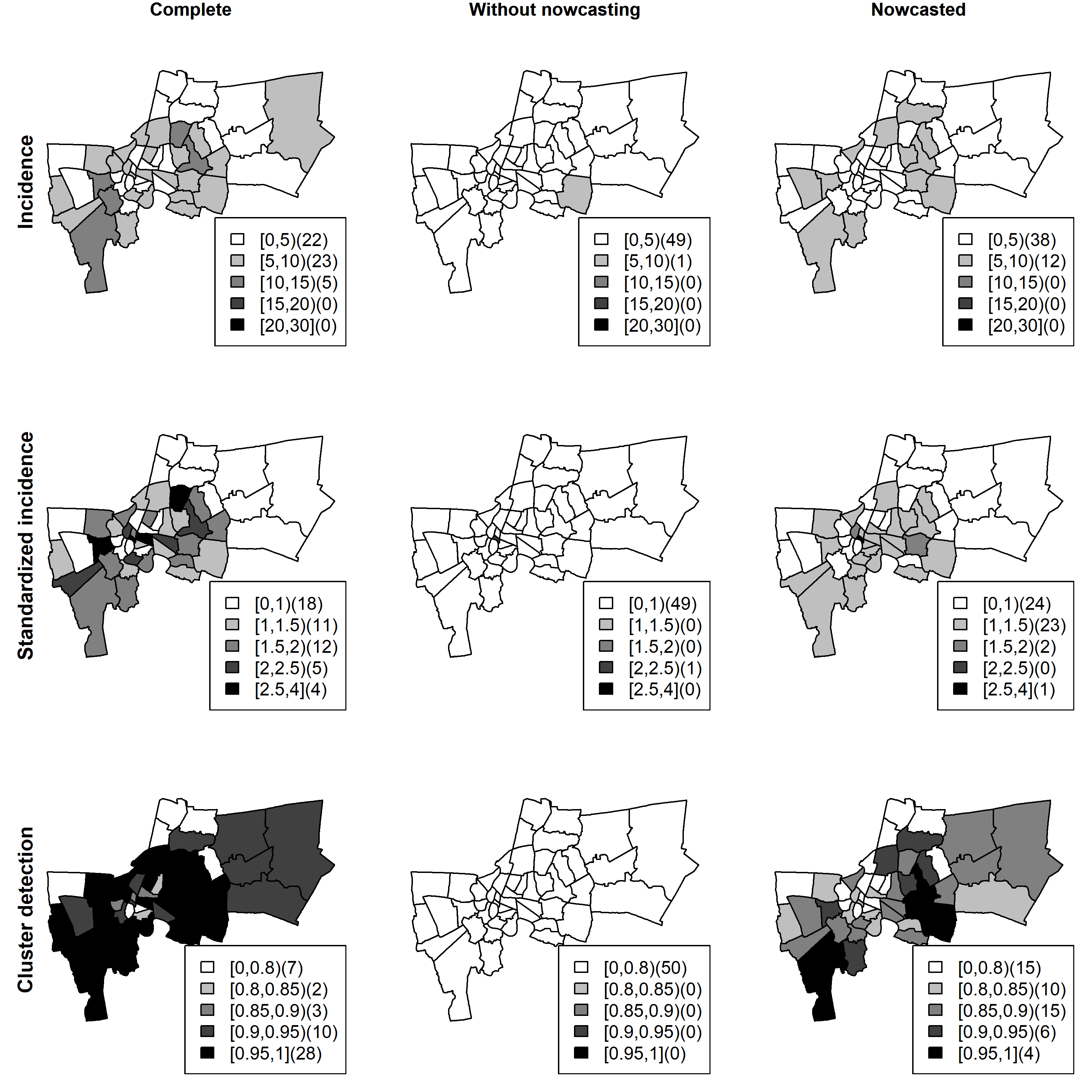
**

**Fig S2** Maps of dengue incidence, standardized incidence and cluster detection using EXC and true cases, with and without nowcasting during week 97.

**
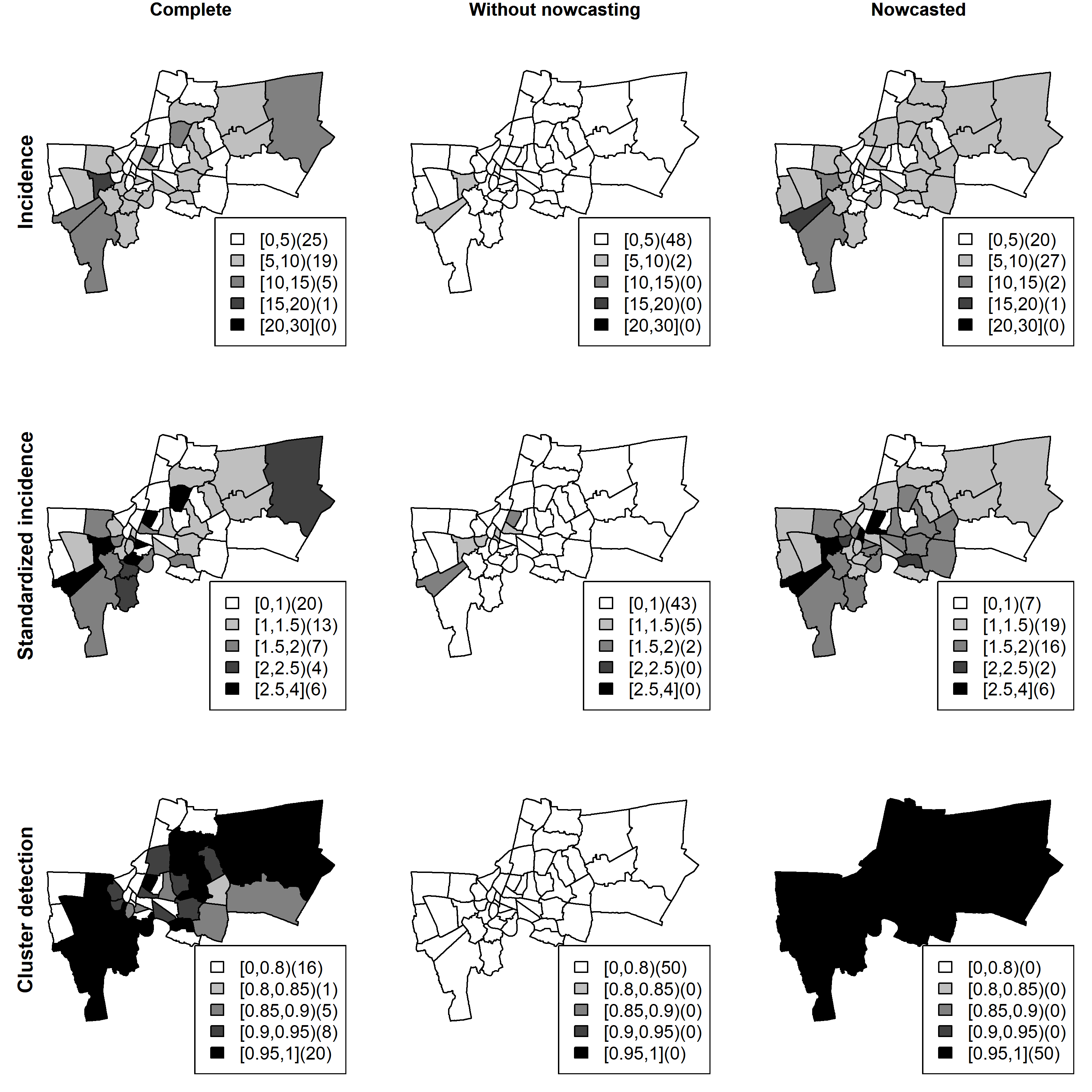
**

**Fig S3** Maps of dengue incidence, standardized incidence and cluster detection using EXC and true cases, with and without nowcasting during week 98.

**
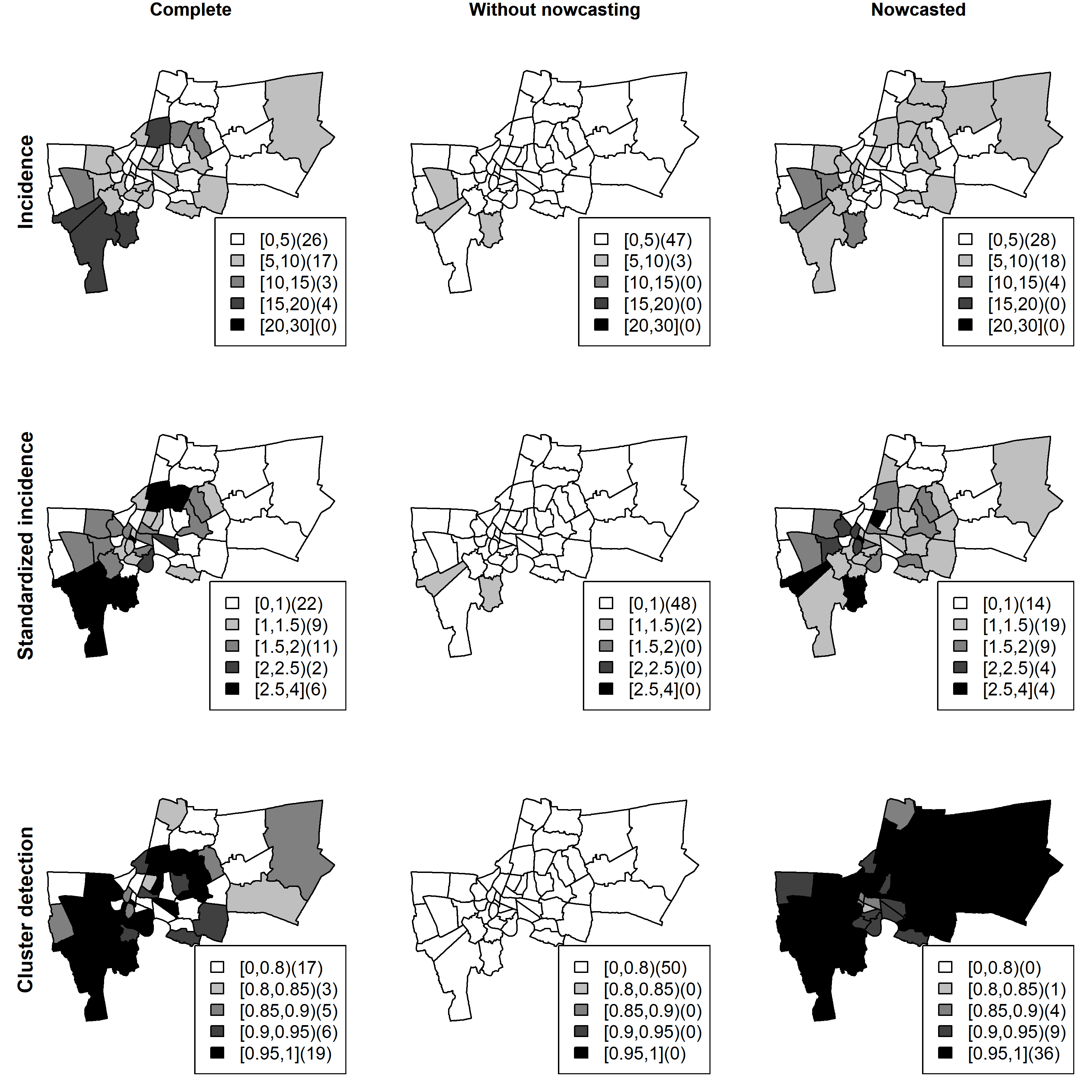
**

**Fig S4** Maps of dengue incidence, standardized incidence and cluster detection using EXC and true cases, with and without nowcasting during week 99.

**
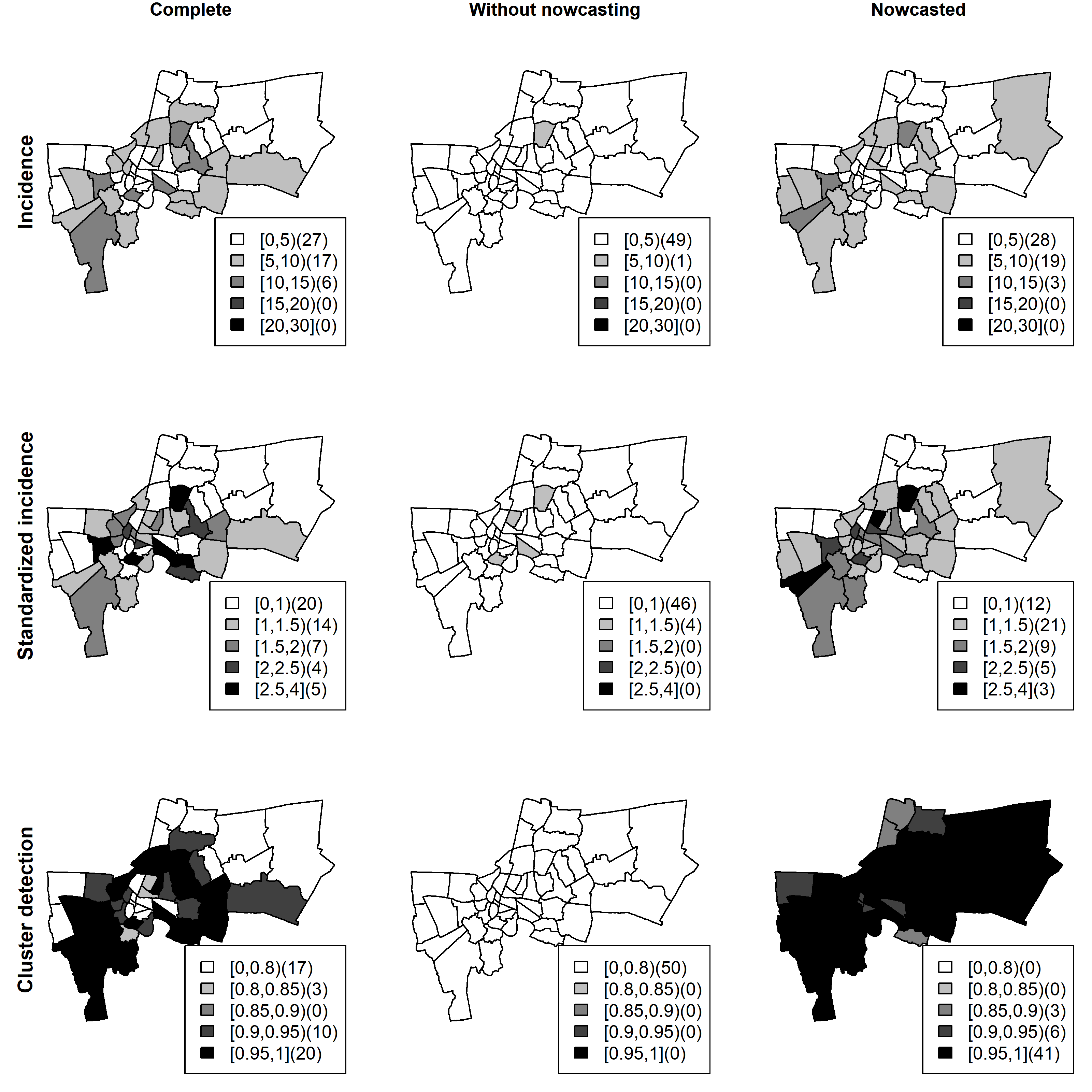
**

**Fig S5** Maps of dengue incidence, standardized incidence and cluster detection using EXC and true cases, with and without nowcasting during week 100.


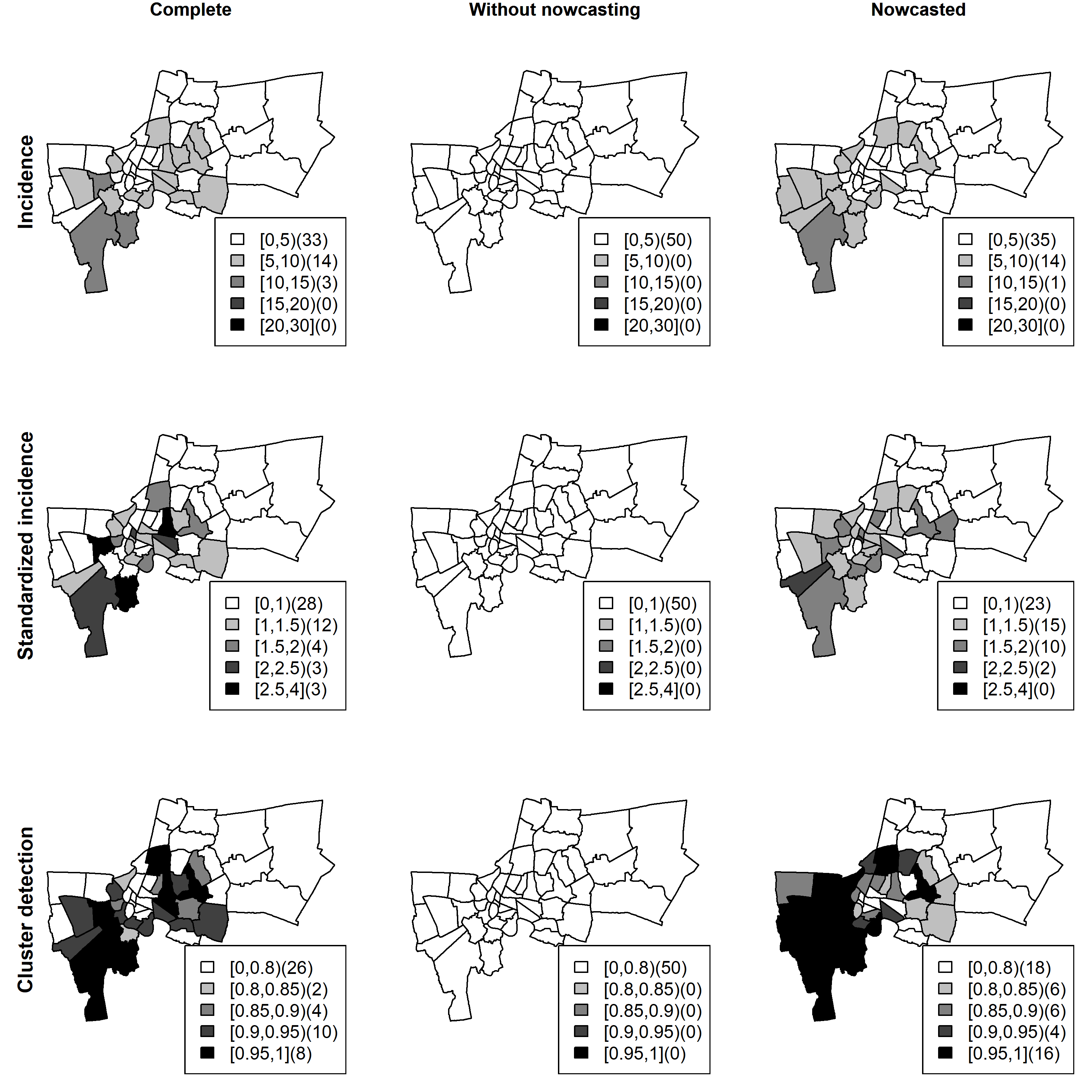


**Fig S6** Maps of dengue incidence, standardized incidence and cluster detection using EXC and true cases, with and without nowcasting during week 101.


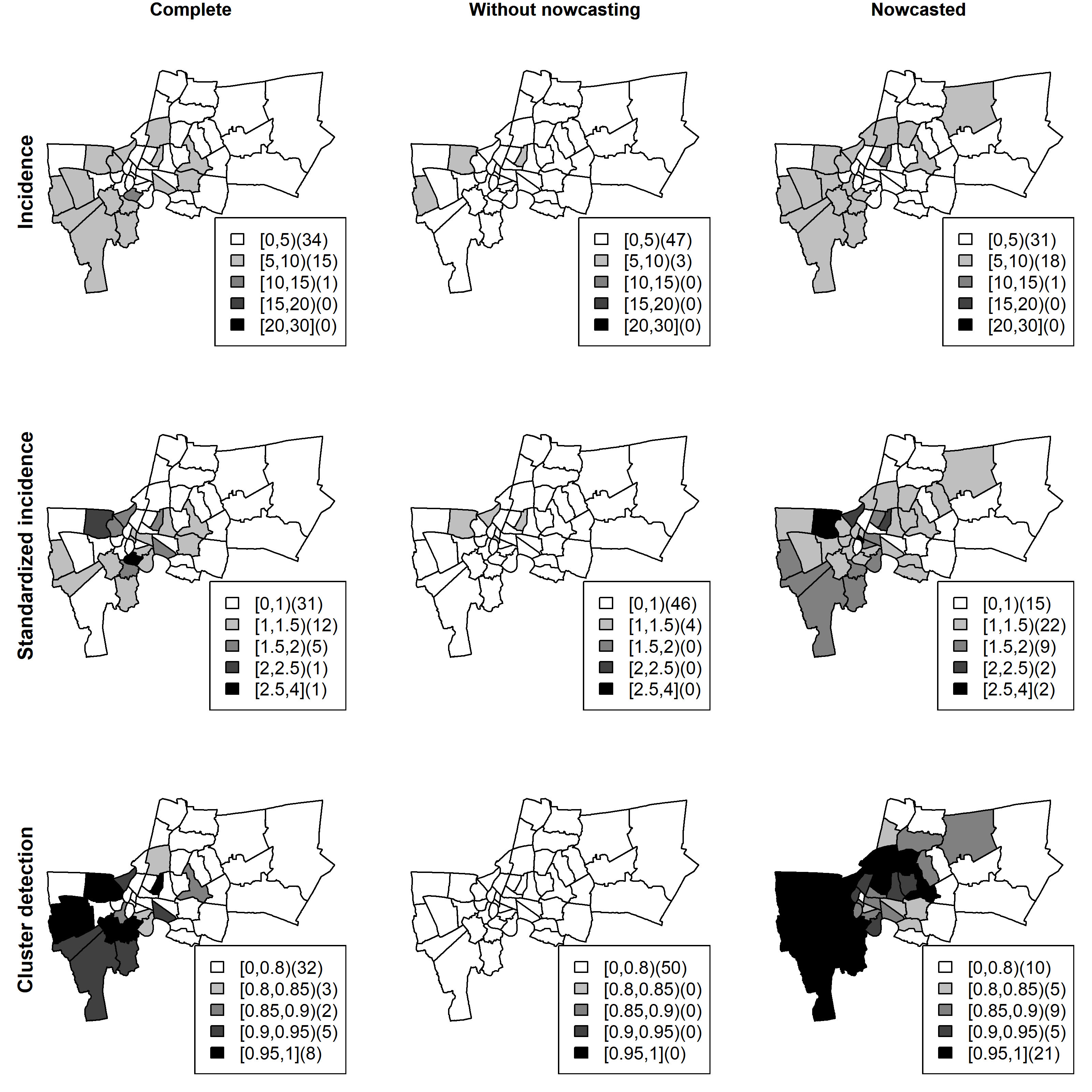


**Fig S7** Maps of dengue incidence, standardized incidence and cluster detection using EXC and true cases, with and without nowcasting during week 102.


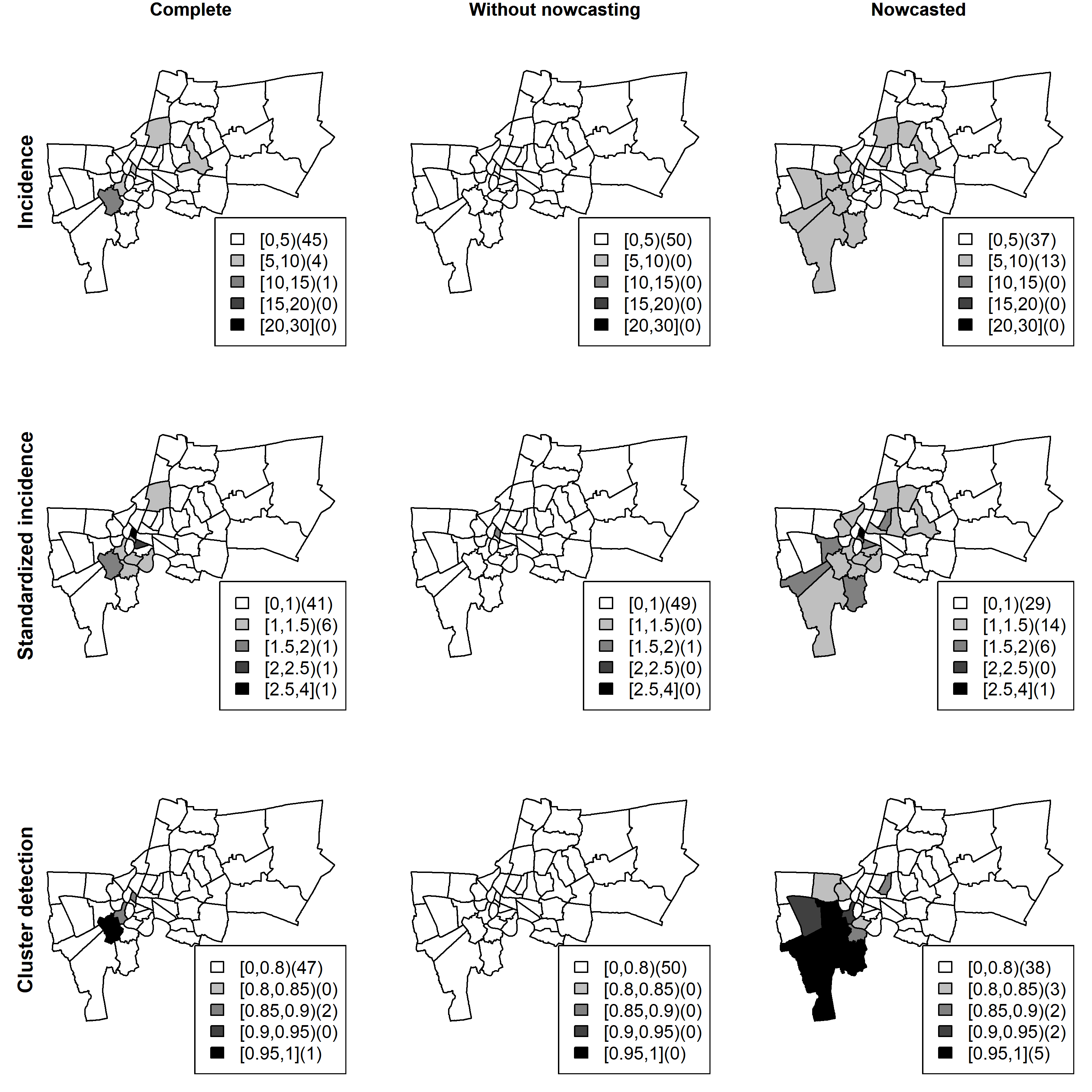


**Fig S8** Maps of dengue incidence, standardized incidence and cluster detection using EXC and true cases, with and without nowcasting during week 103.


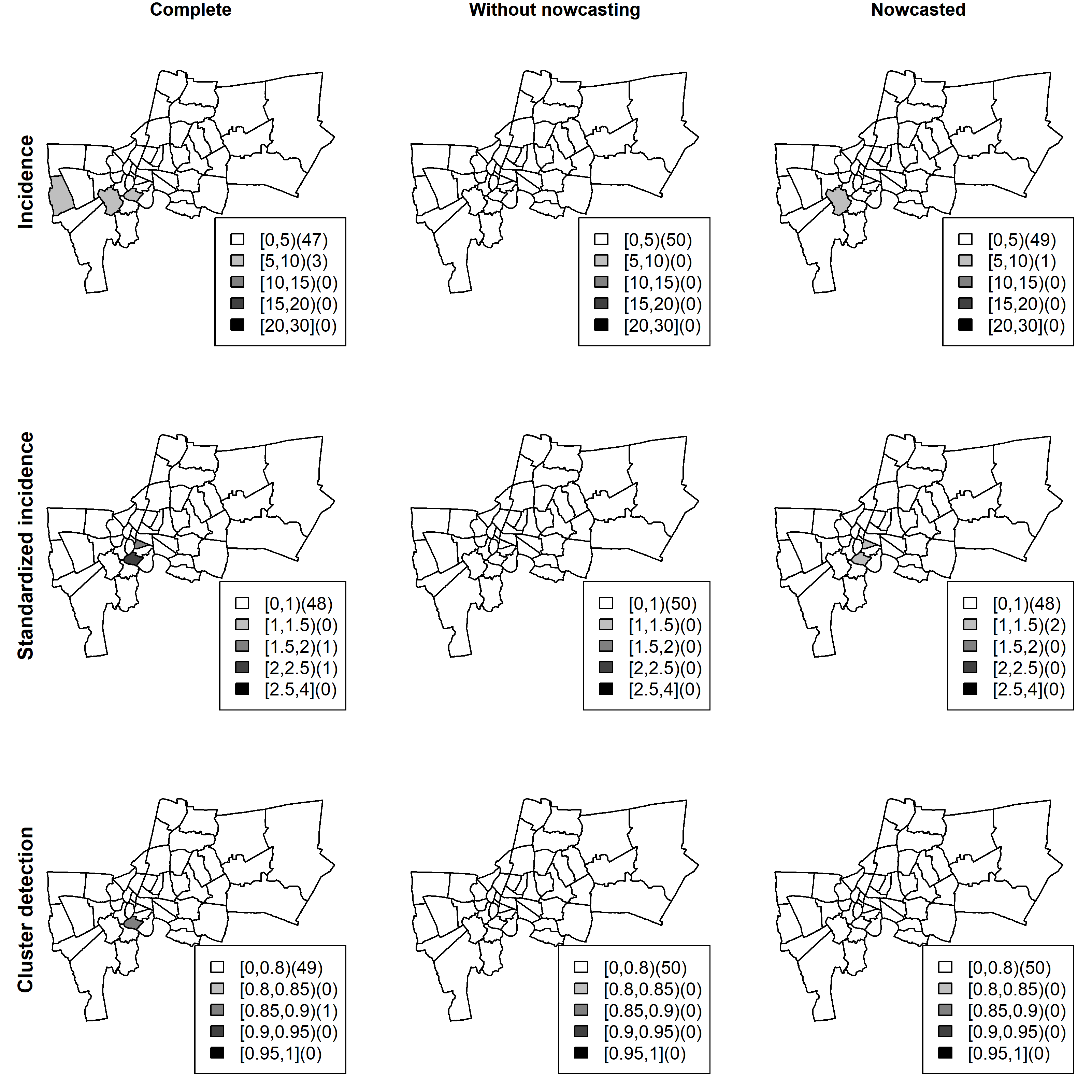


**Fig S9** Maps of dengue incidence, standardized incidence and cluster detection using EXC and true cases, with and without nowcasting during week 104.

**References**

1. Rue, H., S. Martino, and N. Chopin, *Approximate Bayesian inference for latent Gaussian models by using integrated nested Laplace approximations.* Journal of the royal statistical society: Series b (statistical methodology), 2009. **71**(2): p. 319-392.

2. Blangiardo, M. and M. Cameletti, *Spatial and spatio-temporal Bayesian models with R-INLA*. 2015: John Wiley & Sons.

3. Wang, X., Y.R. Yue, and J.J. Faraway, *Bayesian regression modeling with INLA*. 2018: CRC Press.
